# Preparation of the Flexible Green Body of YAG Ceramic Fiber by Melt Spinning

**DOI:** 10.3390/polym14102096

**Published:** 2022-05-20

**Authors:** Hongmei Liu, Junjie Tian, Gangwei Pan, Yongjin Xie, Qing Yao

**Affiliations:** 1 School of Mechanical Engineering, Nantong University, Nantong 226000, China; liu.hm@ntu.edu.cn (H.L.); 2009310006@stmail.ntu.edu.cn (J.T.); xieyongjin1030@163.com (Y.X.); 2 School of Transportation and Civil Engineering, Nantong University, Nantong 226000, China; 3 School of Textile and Clothing, Nantong University, Nantong 226000, China; pangangwei@ntu.edu.cn

**Keywords:** flexibility, YAG ceramic fiber, melt spinning, thermoplastic polyurethane (TPU)

## Abstract

YAG ceramic fiber, with its high thermal conductivity and easy to achieve limit size, provides design flexibility as a laser gain medium. Its mainstream forming method was mainly high-pressure extrusion, but there were disadvantages, such as lack of flexibility. In this work, the flexible green body of YAG ceramic fiber was prepared by melt spinning. The melting characteristics of TPU with four different Shore hardnesses were systematically investigated. The microstructure, element homogeneity of the surface and fracture SEM images of the prepared ceramic fiber were also analyzed in detail. The optimized process parameters of YAG ceramic fiber preparation were as follows: the melting temperature was 220 °C, the screw feed rate of the double-cone screw extruder was F = 15.0 mm/min and the TPU-95A# was used. The ceramic fiber with the mass ratio of TPU-95A# to ceramic powder = 4:6 had the best microstructure quality. It had good flexibility and could be knotted with a bending radius of about 2.5 mm, and the tensile strength reached approximately 20 MPa. These results are crucial for advancing YAG ceramic fiber applications.

## 1. Introduction

Since the invention of the first fiber laser in 1961 [1], fiber laser technology has been developing from low power to high power. Now, the fiber laser is moving in the direction of “any wavelength, any pulse duration and any power”. With its compact structure, high conversion efficiency, convenient thermal management and flexible operation, the fiber laser has been widely used in advanced manufacturing, energy exploration, biomedicine, defense security and other fields [2]. Therefore, the further development of high-power fiber laser technology has become urgent, and it is of great significance. The preparation of the optical gain fiber as one of the core technologies is key.

Due to the low thermal conductivity of quartz glass (1.38 W/(m·K)), poor mechanical performance and large bending radius, the traditional fiber laser is prone to large thermal gradients, optical distortion, limited output power and mechanical damage during long-time operation. Therefore, the application of high-power fiber lasers is limited by the intrinsic properties of quartz glass [3]. Single crystal fibers, such as the Y_3_Al_5_O_12_ (YAG) crystal fiber, have the advantages of excellent physical and chemical properties of crystal and thermal management. They can meet the application requirements of high-power lasers [4]. In 2012, a French research team used the Yb: YAG single fiber prepared by micro-pull-down to achieve the 251 W maximum continuous laser output power and 53% slope efficiency at 1030 nm, which is the highest continuous laser output power and the highest conversion efficiency obtained with the single crystal fiber thus far [5]. Researchers of the Chinese Academy of Sciences have recently developed a new laser heating base (LHPG) single crystal optical fiber growth furnace. They have successfully prepared a Yb: YAG single crystal optical fiber with a diameter of 0.2 mm and a length of 710 mm. It has the length–diameter ratio of a single crystal fiber >3000 and the diameter fluctuates within ±5%, with the highest aspect ratio among similar single crystal fibers in China [6]. Nevertheless, the preparation temperature of a single crystal optical fiber is generally above the melting point. It has a complex production process, high equipment requirements, high energy consumption and high cost. Especially, due to the separation coefficient of a single crystal, high concentration doping is hindered, resulting in limited power improvement [3].

In 1995, Dr. Akio Ikesue prepared the world’s first laser transparent ceramics [7]. Transparent ceramics, with their high concentration of uniform doping, high thermal conductivity and easy to achieve limit size, provide design flexibility as a laser gain medium [8]. Accordingly, Kim and Fair from the U.S. Air Force Laboratory successfully realized the preparation of a 30 μm diameter ceramic fiber using transparent ceramic materials, verifying the feasibility of the fiber laser as a gain medium [9]. The materials used in ceramic fiber are the most representative in yttrium aluminum garnet structure (Y_3_Al_5_O_12_, YAG) transparent ceramics, with a wide range of uses, good optical performance and high quantum efficiency advantages. Their good performance is well adapted to the requirements of a high-power optical fiber laser [10,11,12]. For instance, Ikesue prepared the Nd: YAG ceramic fiber that was 65 mm in length and 900 m in diameter by extrusion and solid phase reaction [13,14]. Kim and Fair extruded a YAG fiber green body through a 125 mm nozzle and sintered a highly transparent YAG transparent ceramic fiber with hot isostatic pressure. The ceramic fiber diameter was about 20 μm and bend radius was 3 mm [15,16,17]. Recently, a novel route that combined aqueous gelcasting with a capillary glass tube was designed to prepare a Yb: YAG transparent ceramic fiber with a diameter of 1.0 mm and length of 43.0 mm for the first time [18].

In addition to laser applications, YAG ceramic fiber is also of great interest in scintillator research [19]. Dai and Wu prepared Ce: LuAG scintillation single crystal fibers (SCFs) with excellent scintillation performance by adopting the laser-heated pedestal growth (LHPG) method [20]. YAG ceramic fiber even has broad prospects in nuclear/fusion applications [21]. Under different temperature radiation, the track diameters in both YAP and YAG were very similar, and less energy was required for YAG [22].

However, due to the micron diameter, the preparation of YAG ceramic fiber was difficult. Its mainstream forming method was mainly the high-pressure extrusion of Kim and Fair and Ikesue et al. [16,23,24]. However, there are disadvantages to extrusion such as metal ion impurities, organic matter residue and lack of flexibility. Therefore, it is very necessary to explore new forming methods to overcome the shortcomings.

In this paper, a flexible green body of YAG transparent ceramic fiber was prepared by the melt-spinning process for the first time. The process parameters under TPU with four different Shore hardnesses, screw feed rates and melting temperatures were systematically studied. The microstructure and uniformity of the green body were fully analyzed. In particular, the mechanical properties such as strength, flexibility and bending radius were researched in detail. The flexibility and bending strength of the YAG ceramic fiber were significantly improved.

## 2. Experimental Procedure

**Figure 1:**
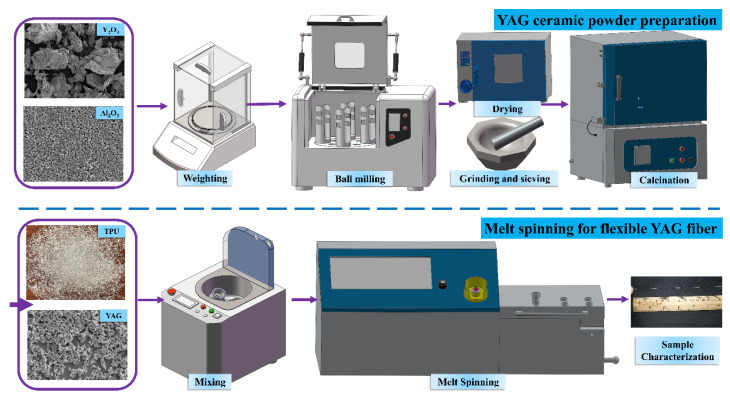
Process of preparing the flexible green body of YAG ceramic fiber by melt spinning.

The preparation process for the flexible green body of YAG ceramic fiber is shown in Figure 1. It mainly consisted of two parts: the preparation of YAG ceramic powder and the preparation of the flexible green body of YAG fiber by melt spinning.

### 2.1. YAG Ceramic Powder Preparation

In the present study, Y_2_O_3_ (99.99% purity, Yuelong New Materials Co., Ltd., Shanghai, China), with an average particle size of 6.0 μm, and α-Al_2_O_3_ (99.99% purity, Shanghai Yuelong Chemical Co., Ltd., Shanghai, China), with the mean particle size of 160.0 nm, were used as starting materials. The above-mentioned powders with good dispersion and uniform were weighed based on the stoichiometric ratio of Y_3_Al_5_O_12_. The sintering additives were tetraethyl orthosilicate (TEOS, 99.99% purity, Alfa Aesar, Ward Hill, Haverhill, MA, USA) with an amount of 0.5 wt.% and MgO (99.99% purity, Alfa Aesar, Ward Hill, Haverhill, MA, USA) with an amount of 0.1 wt.%. They were mixed with absolute ethyl alcohol and then ball milled at a rotation speed of 200 r/min using high-purity Al_2_O_3_ balls. After 12 h of ball milling, the mixed powders had good uniformity and the average particle size was 360 nm. Afterwards, the milled slurry was dried, ground and sieved through a 200-mesh screen three times. Then, the mixed powders were calcined at 800 °C for 8 h in a muffle furnace.

### 2.2. Melt Spinning for Flexible Green Body

**Figure 2:**
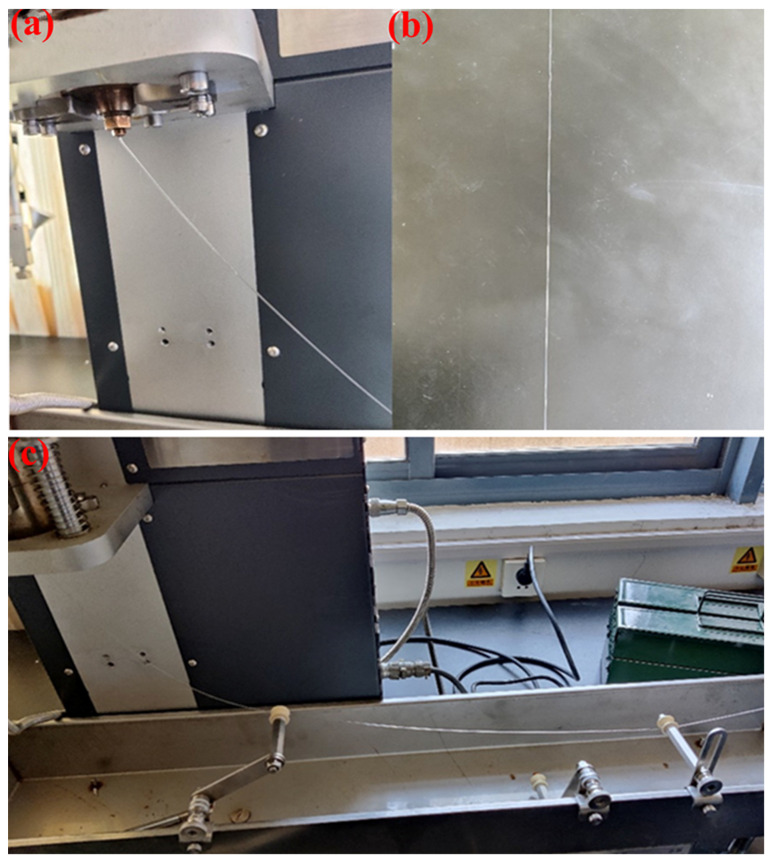
Equipment for melt spinning and drawing: (**a**) nozzle of melt spinning machine, (**b**) YAG ceramic fiber cooling in air, (**c**) drawing mechanism and forming process.

The commercial thermoplastic polyurethane (TPU) particles of different Shore hardnesses 75A, 80A, 90A and 95A were labelled TPU-75A#, TPU-80A#, TPU-90A# and TPU-95A#, respectively. They were thoroughly mixed with the prepared YAG ceramic powders in a mixer (HWV 800, Shenzhen Hasai Technology Co., Shenzhen, China) at a speed of 100 r/min. The mass ratio of TPU to YAG ceramic powder was 4:6 or 3:7. Then, the ceramic/TPU mixes were put into a miniature twin-cone screw extruder (WLG10G, Shanghai Xinshuo Ltd., Shanghai, China) and a matching drawing machine for melt spinning (shown in Figure 2). The twin-cone screw extruder was set at different screw feed speeds (5 mm/min, 10 mm/min, 15 mm/min and 20 mm/min), different melt temperatures (160 °C, 180 °C, 200 °C and 220 °C), and different nozzle diameters (0.2 mm, 0.3 mm and 0.5 mm) (Figure 2a). Meanwhile, the extruded fibers were cooled in air (Figure 2b) and drawn continuously by a drawing machine (Figure 2c). Finally, the continuous and flexible green body of YAG ceramic fiber was obtained successfully by melt spinning.

### 2.3. Characterizations

The surfaces and fracture of the YAG fiber green body and the distribution of elements in the YAG fiber were observed by a scanning electron microscope (SEM, JSM-6510, JEOL, Kariya, Japan) with an energy dispersive spectrometer (EDS, Aztec, Oxford Instruments, Oxford, UK) system.

The flow rate of melt mass of TPUs with different Shore hardnesses was determined by a melt index meter (XNR-400C, Goettfert, Germany). The TPU particles were melted into a plastic fluid at a melting temperature of 205 °C and a load of 2.16 kg. This was followed by the mass exiting through a 2.1 mm diameter circular tube within 10 min. The greater the mass, the better the melt flow of the TPU, and vice versa.

The diameter and tensile strength of YAG ceramic fiber with different components were tested by a monofilament strength meter with a loading rate of 2 mm/min (YM-06D, Nantong Hongda Experimental Instrument Co., Ltd., Nantong, China).

## 3. Results and Discussion

TPU has excellent properties of good elasticity and high strength. The melting characteristics of the organic polymer can be used as the “binder” of inorganic ceramic powder. More importantly, TPU, with both excellent mechanical properties and melt fluidity, can meet the forming demand of YAG ceramic fiber; thus, the prepared YAG ceramic fiber also had high strength and good flexibility [25,26]. Therefore, TPU, with its high transparency and purity, and different Shore hardnesses of 75A, 80A, 90A and 95A, was selected in this study [27,28].

**Figure 3:**
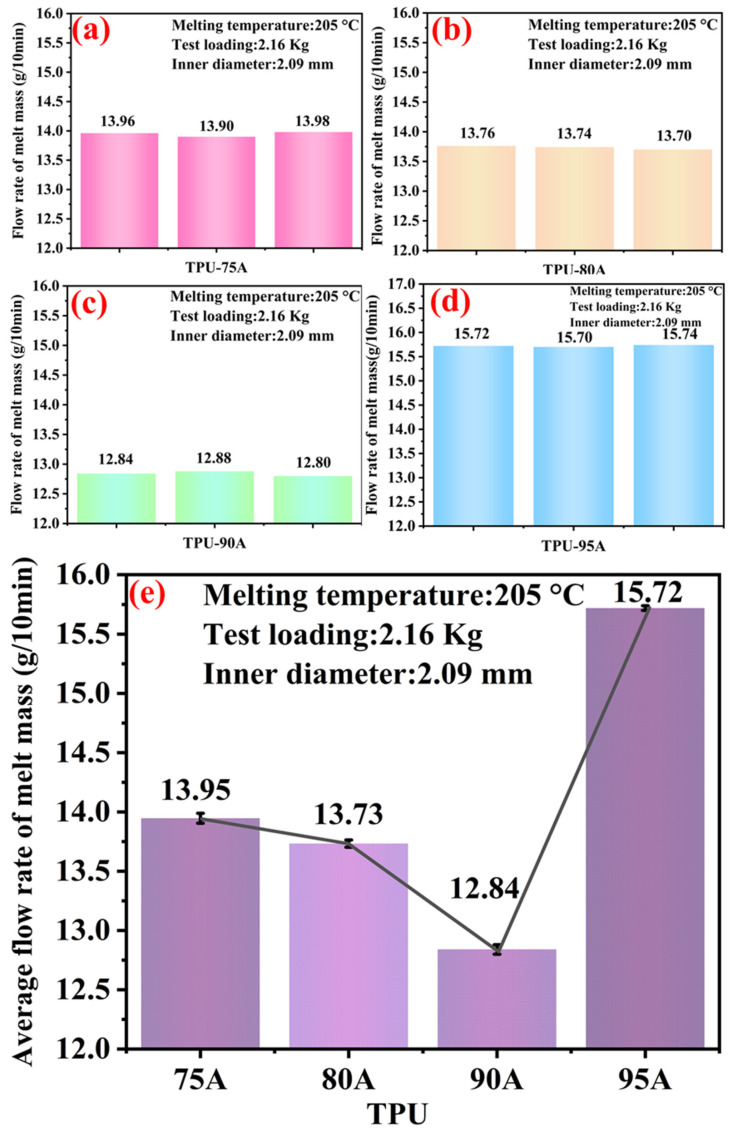
Histograms of flow rate of melt mass and average flow rate of melt mass for different TPUs: (**a**) TPU-75A#, (**b**) TPU-80A#, (**c**) TPU-90A#, (**d**) TPU-95A#; (**e**) average flow rate of four TPUs’ melt mass.

Figure 3 shows the flow rate of melt mass and average flow rate of melt mass for TPU with different Shore hardnesses under the same experimental conditions. Figure 3a shows that the melt mass flow rate of TPU-75A# was 13.96 g/10 min, 13.90 g/10 min and 13.98 g/10 min. Figure 3b displays the melt mass flow rate of TPU-80A# as 13.76 g/10 min, 13.74 g/10 min and 13.70 g/10 min. In Figure 3c, the melt mass flow rates of TPU-90A# were 12.84 g/10 min, 12.88 g/10 min and 12.80 g/10 min. Lastly, in Figure 3d, it was found that the melt mass flow rates of TPU-95A# were 15.72 g/10 min, 15.70 g/10 min and 15.74 g/10 min. According to Figure 3a–d, it can be described that the melt mass flow rate error of the four-hardness TPUs is very small after three tests. Consequently, it could be expressed by the average flow rate of the corresponding melt mass. The average flow rates were 13.95 g/10 min, 13.73 g/10 min, 12.84 g/10 min and 15.72 g/10 min of TPU-75A#, TPU-80A#, TPU-90A#, and TPU-95A#, respectively, as shown in Figure 3e. Obviously, TPU-95A# had the highest average melt mass flow rate. TPU-90A# had a better hardness value, but the melt mass flow rate was not as good as TPU-75A# and TPU-80A#. It could be seen that the Shore hardness was not positively correlated with the flow rate of melt mass, and TPU-95A # had the best hardness and melting characteristics in this experiment.

To further select the appropriate TPUs and optimize the process parameters of the miniature twin-cone screw extruder, the performance of ceramic fiber under different TPU types, screw feed rates and melting temperatures was tested by a single variable method to obtain the best melt-spinning process conditions. Moreover, the melting rate, flowability, formation of silk of molten mass and fiber tensile strength and flexibility were set as “perceptual observation characteristics”. Table 1 presents the concerned properties, symbols and meanings of the molten mass and YAG ceramic fibers. Among them, the speed of the melting rate is represented by the number of pentagrams, the intensity of tensile strength is expressed by the number of circles, the difference in flexibility is represented by the number of squares, the fluidity of the molten body is represented by the number of triangles and the difficulty of filamentation is represented by the number of rhombuses.

Table 2 provides the performance effects of different TPUs on ceramic fiber at the melting temperature of 220 °C, feed speed of 15 mm/min and a nozzle diameter of 0.3 mm. All four TPU types could melt and spin smoothly, but the TPU-95A# ceramic fiber had the best melting rate, tensile strength and flexibility. The TPU-95A# served as the best TPU option accordingly.

Table 3 demonstrates the performance effects of the different feed speeds of screws on the ceramic fiber with a melting temperature of 220 °C, TPU-95A# and a nozzle diameter of 0.3 mm. It was found that within a certain range (from 5 mm/min to 15 mm/min), improving the feed speed not only accelerated the melting rate, but also promoted the tensile strength and flexibility of the ceramic fiber. Nevertheless, when the feed speed exceeded a certain value and reached 20 mm/min, the increased shear rate would destroy the molecular chain in the TPU [29,30]. It gradually untied and slid the molecules from the network structure, and decreased the concentration of the physical crosslinking point, leading to further improvement of the melting rate [31,32]. However, the excessive reduction of molecular weight reduced the mechanical properties of TPU, which reduced the flexibility and elasticity of the finished product. Therefore, the feed speed of the optimized screw was 15 mm/min.

Table 4 displays that the influence of different melting temperatures on the performance of YAG ceramic fiber under the conditions of TPU-95A#, a screw feed speed of 15 mm/min and a nozzle diameter depending on the situation. It was clearly found that with the temperature increased from 160 °C to 220 °C, the TPU melting rate and fluidity gradually improved and the formation by melt spinning also went from difficult to easy. In addition, the optional nozzle diameter could also be smaller and smaller. The melting temperature of 220 °C met the requirements for preparing the YAG ceramic fiber in this experiment, and hence, 220 °C was preferred as the optimized temperature parameter. The higher melting temperature would have damaged the service life of the experimental equipment; as a result, it was not tested.

Through the above series of experiments, it was found that the optimized process parameters of YAG ceramic fiber preparation were as follows: the melting temperature was 220 °C, the screw feed rate of the micro double-cone screw extruder was F = 15.0 mm/min and TPU-95A# was used.

**Figure 4:**
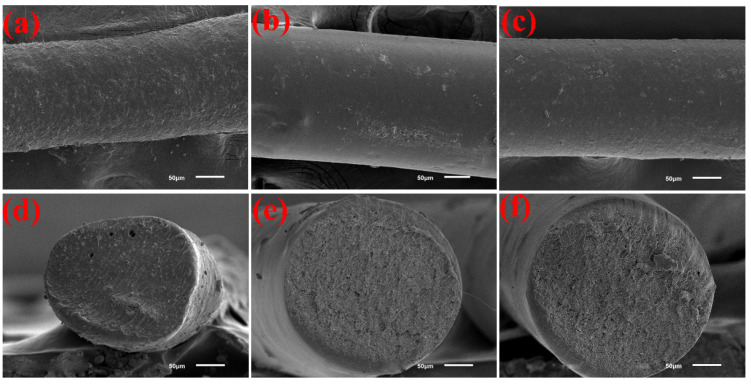
SEM images of the surface and fracture of YAG ceramic fiber with different TPU types and the ratios of TPU to ceramic powder: (**a**,**d**) surface and fracture of the ceramic fiber with the mass ratio of TPU-75A# to ceramic powder = 4:6, respectively; (**b**,**e**) surface and fracture of the ceramic fiber with the mass ratio of TPU-95A# to ceramic powder = 4:6, respectively; (**c**,**f**) surface and fracture of the ceramic fiber with the mass ratio of TPU-95A# to ceramic powder = 3:7, respectively.

Figure 4 shows the SEM image of the surface and fracture of the YAG ceramic fiber with different TPU types and the ratios of TPU to ceramic powder. Figure 4a,b reveals the SEM image of the surface and fracture of the ceramic fiber with the mass ratio of TPU-75A# to ceramic powder = 4:6, respectively. The comparison of SEM images shows that the surface of ceramic fiber in Figure 4a is coarser than that in Figure 4b. This was because the hardness of TPU-75A# was lower than that of TPU-95A#. The preparation of the ceramic fiber was through the nozzle, forming the silk through plastic deformation; the plastic deformation was mainly caused by the resistance of the TPU molecular chain to the friction shear force [33]. In other words, the higher the hardness of TPU, the more conducive it was to resist friction and shear force [34,35]. This would make the wear of the ceramic fiber surface lower, and the prepared ceramic fiber surface smoother. Accordingly, it can be seen from Figure 4d that when the melting index and hardness of TPU-75A# were low, the fracture SEM image of the prepared ceramic fiber had microporosity. This led to a reduction in density and strength of the ceramic fiber.

The comparison between Figure 4b,c shows that the surface of the ceramic fiber in Figure 4b is smoother than that in Figure 4c; that is, the surface of the ceramic fiber with the mass ratio of TPU-95A# to ceramic powder = 3:7 is rougher (as shown in Figure 4c). This was because the ceramic powder used in this experiment was mainly composed of Y_2_O_3_ and Al_2_O_3_. Excessive metal oxide powder absorbs heat at a high temperature. As a result, the melting of the TPU polymer was not sufficient for it to remain on the surface of the fiber, resulting in a rough surface on the ceramic fiber.

Accordingly, by comparing Figure 4e,f, it can be found that the SEM fracture of Figure 4e was smoother than that of Figure 4f. Both are more complete and nonporous than in Figure 4d, mainly because the hardness and melting characteristics of TPU-95A# were better than TPU-75A#, which coincides with the conclusion of Figure 3. In addition, the SEM fracture of the ceramic fiber in Figure 4f seemed to have a certain incomplete molten sheet of TPU-95A#. This indicated that with the increase in the mass ratio of ceramic powder, the powder was not only not able to be completely wrapped by the melting TPU, but also easily led to insufficient TPU melting [36,37]. The prepared ceramic fiber was not as high in quality as the quality in Figure 4e.

Therefore, according to the surface and fracture SEM images in Figure 4, the ceramic fiber with the mass ratio of TPU-95A# to ceramic powder = 4:6 has the best microstructure quality.

**Figure 5:**
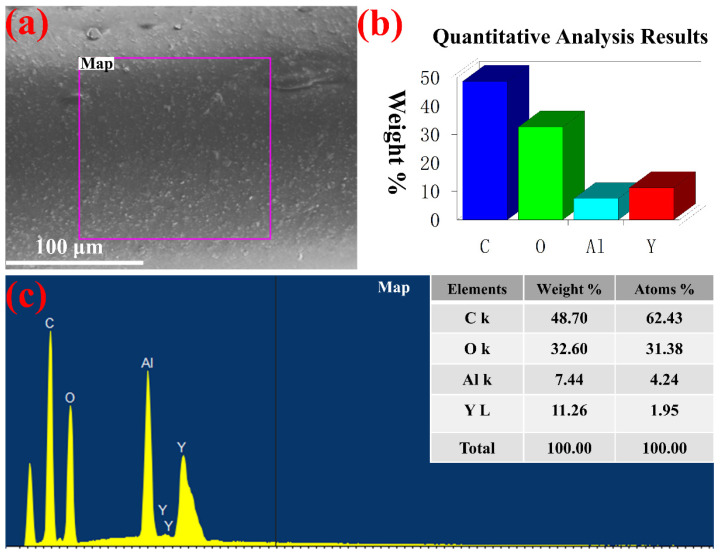
EDS analysis of the ceramic fiber (TPU-95A#, the mass ratio of TPU-75A# to ceramic powder = 4:6): (**a**) surface SEM image from surface scanning of ceramic fiber, (**b**) histogram of elemental quantitative analysis, (**c**) plot of types and intensities of elements detected (embedded table: the mass and atomic specific gravity of the four elements).

Figure 5 demonstrates the EDS analysis of the ceramic fiber (TPU-95A#, the mass ratio of TPU-75A# to ceramic powder = 4:6). Figure 5a shows the surface SEM image from the surface scanning of the ceramic fiber. Figure 5b displays the histogram of the elemental quantitative analysis, and Figure 5c plots the types and intensities of elements detected (embedded table: the mass and atomic specific gravity of the four elements). In Figure 5c, the weight percentages of the C, O, Al and Y elements were 48.70, 32.60, 7.44 and 11.26, respectively, and the atomic percentages were 62.43, 31.38, 4.24 and 1.95, respectively. The sum of the weight percentage and the atomic percentage of each element was 100. Among them, C, O and undetected H elements were mainly provided by the polymer TPU, whereas Al, Y and O elements were provided by the Y_2_O_3_ and Al_2_O_3_ ceramic powder, with no other elements. It can be inferred that there were no other impurity elements in the preparation process of the ceramic fiber in this experiment. The process of melt spinning adopted in this study can prepare the high-quality and pure ceramic fiber.

**Figure 6:**
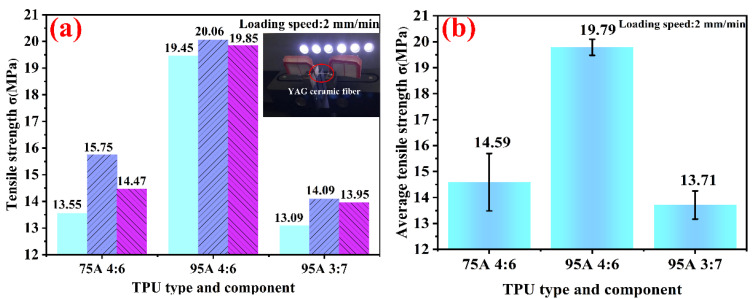
Tensile strength of different TPU types and components: (**a**) three tensile strength tests of different TPU types and components (TPU-75A# and ceramic powder = 4:6, TPU-95A# and ceramic powder = 4:6, TPU-95A# and ceramic powder = 3:7; inset: the test situation by the monofilament strength tester), (**b**) average tensile strength of different TPU types and components.

Figure 6 demonstrates the tensile strength of different TPU types and components. The tensile strength of the ceramic fiber was measured at a loading rate of 2 mm/min in a monofilaments strength tester. Figure 6a shows that the tensile strengths of TPU-75A# and ceramic powder = 4:6 were 13.55 MPa, 15.75 MPa and 14.47 MPa. The tensile strengths of TPU-95A# and ceramic powder = 4:6 were 19.45 MPa, 20.06 MPa and 19.85 MPa. The tensile strengths of TPU-95A# and ceramic powder = 3:7 were 13.09 MPa, 14.09 MPa and 13.95 MPa. The histogram of two groups (TPU-75A# and ceramic powder = 4:6 and TPU-95A# and ceramic powder = 4:6) shows that under the condition of a constant mass ratio of ceramic powder, the tensile strength of ceramic fiber made by TPU-95A# with a high hardness was higher than that made by TPU-75A# with a low hardness. It further confirmed that for TPU, having a high hardness improves the toughness and tensile strength of ceramic fiber. In addition, the histogram of two groups (TPU-95A# and ceramic powder = 4:6 and TPU-95A# and ceramic powder = 3:7) displays that when the TPU hardness was the same, the tensile strength decreased significantly with the decrease in the TPU ratio. Excessive metal oxides in ceramic powders may also lead to the incomplete melting of TPU. 

Figure 6b exhibits the average tensile strength calculated based on Figure 6a from the results of TPU-75A# and ceramic powder = 4:6, TPU-95A# and ceramic powder = 4:6 and TPU-95A# and ceramic powder = 3:7. Their average tensile strengths were 14.59 MPa, 19.79 MPa and 13.71 MPa, respectively. Apparently, the average tensile strength of TPU-95A# and ceramic powder = 4:6 was larger than that of the other TPU types and components, and the error fluctuation was smaller than that of the others. Therefore, Figure 6 adequately shows that the ceramic fiber with TPU-95A# and ceramic powder = 4:6 had the best flexibility and uniformity, which was also consistent with the microstructure of Figure 4.

**Figure 7:**
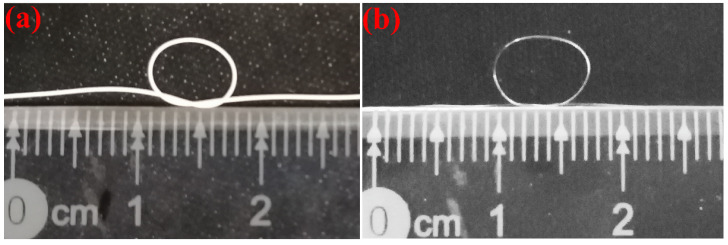
Sample of YAG ceramic fiber by melt spinning: (**a**) flexible green body of ceramic fiber with the mass ratio of TPU-95A# to ceramic powder = 4:6, (**b**) TPU-95A# melt spinning.

Figure 7 shows a sample of the YAG ceramic fiber prepared by melt spinning. Figure 7a displays the flexible green body of YAG ceramic fiber with the mass ratio of TPU-95A# to ceramic powder = 4:6. This green body had good flexibility and could be bent and knotted with a bending radius of about 2.5 mm. Figure 7b shows the fiber prepared by TPU-95A#, with a bending radius of about 2.5 mm. Therefore, melt spinning was an effective way to prepare ceramic fiber with high flexibility and high strength. In the future, further research on the sintering and polishing of ceramic fiber will be carried out.

## 4. Conclusions

In this study, the flexible ceramic fiber was prepared by melt spinning. The melting characteristics with TPUs and four different Shore hardnesses were systematically investigated. The tensile strength and flexibility of ceramic fiber was tested. The microstructure, element types and homogeneity of the surface and fracture SEM images were also analyzed in detail. Through the above series of experiments, the optimized process parameters of YAG ceramic fiber preparation were as follows: the melting temperature was 220 °C, the screw feed rate of the micro double-cone screw extruder was F = 15.0 mm/min and the TPU-95A# was used. In addition, according to the surface and fracture SEM images, the ceramic fiber with the mass ratio of TPU-95A# to ceramic powder = 6:4 had the best microstructure quality, and there were no other impurity elements. The resulting flexible green body of YAG ceramic fiber had good flexibility and could be bent and knotted with a bending radius of about 2.5 mm, and the tensile strength reached approximately 20 MPa. Therefore, melt spinning provided a novel path to prepare the green body of YAG ceramic fiber. The popular application and development of ceramic fibers in lasers can be greatly enhanced by this new preparation method.

## Figures and Tables

**Table 1:** Concerned properties, symbols and meanings of the molten mass and YAG ceramic fibers.

Property	Symbols and Meanings
Melting rate	☆ (slow) → ☆☆☆ (fast)
Tensile strength	○ (low) → ○○○ (high)
Flexibility	□ (poor) → □□□ (good)
Flowability	△ (poor) → △△△ (good)
Forming silk	◇ (difficulty) → ◇◇◇ (easy)

**Table 2:** Performance effects of different TPUs on ceramic fiber at melting temperature of 220 °C, feed speed of 15 mm/min and nozzle diameter of 0.3 mm.

No.	TPU Label	Samples Exhibition	Characteristics and Analysis
1	75A	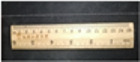	Melting rate: ☆☆ Tensile strength: ○○ Flexibility: □
2	80A	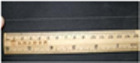	Melting rate: ☆☆ Tensile strength: ○○ Flexibility: □
3	90A	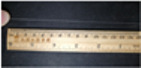	Melting rate: ☆ Tensile strength: ○○○ Flexibility: □□
4	95A	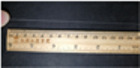	Melting rate: ☆☆☆ Tensile strength: ○○○ Flexibility: □□□

**Table 3:** Performance effects of different feed speeds of screws on ceramic fiber at melting temperature of 220 °C, TPU-95A# and nozzle diameter of 0.3 mm.

No.	Feed Speed	Samples Exhibition	Characteristics and Analysis
1	5	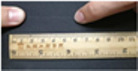	Melting rate: ☆ Tensile strength: ○ Flexibility: □
2	10	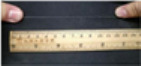	Melting rate: ☆☆ Tensile strength: ○○ Flexibility: □□
3	15	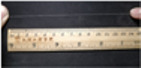	Melting rate: ☆☆☆ Tensile strength: ○○○ Flexibility: □□□
4	20	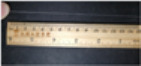	Melting rate: ☆☆☆ Tensile strength: ○ Flexibility: □□

**Table 4:** Performance effects of different melting temperatures on ceramic fiber at feed speed of screw of 15 mm/min, TPU-95A# and nozzle diameter depending on the situation.

No.	Temperature	Samples Exhibition	Characteristics and Analysis
1	160 °C	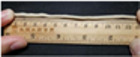	Melting rate: ☆ Flowability: △ Forming silk: ◇
2	180 °C	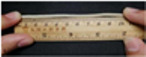	Melting rate: ☆ Flowability: △ Forming silk: ◇
3	200 °C	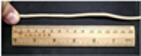	Melting rate: ☆☆ Flowability: △△ Forming silk: ◇◇
4	220 °C	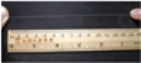	Melting rate: ☆☆☆ Flowability: △△△ Forming silk: ◇◇◇

## Data Availability

Data will be made available from the corresponding authors on reasonable request.

## References

[1] Snitzer E. (1961). Proposed Fiber Cavities for Optical Masers. J. Appl. Phys..

[2] Thomas G.A., Shraiman B.I., Glodis P.F., Stephen M.J. (2000). Towards the clarity limit in optical fibre. Nature.

[3] Fair G.E., Hay R.S., Lee H.D., Boakye E.E., Parthasarathy T.A. (2010). Towards optical quality yttrium aluminum garnet (YAG) fibers: Recent efforts at AFRL/RX. Laser Technol. Déf. Secur. VII.

[4] Wang T., Zhang J., Zhang N., Wu B., Wang S., Jia Z., Tao X. (2019). Research Progress in Preparation of Single Crystal Fiber and Fiber Lasers. Laser Optoelectron. Progr..

[5] Délen X., Piehler S., Didierjean J., Aubry N., Voss A., Ahmed M.A., Graf T., Balembois F., Georges P. (2012). 250 W single-crystal fiber Yb: YAG laser. Opt. Lett..

[6] Wang Y., Wang S., Wang J., Zhang Z., Zhang Z., Liu R., Zu Y., Liu J., Su L. (2020). High-efficiency ∼2 µm CW laser operation of LD-pumped Tm3+: CaF2 single-crystal fibers. Opt. Express.

[7] Ikesue A., Kamata K., Yoshida K. (1995). Synthesis of Nd3+, Cr3+-codoped YAG ceramics for high-efficiency solid-state lasers. J. Am. Ceram. Soc..

[8] Ikesue A., Aung Y.L., Kamimura T., Honda S., Iwamoto Y. (2018). Composite Laser Ceramics by Advanced Bonding Technology. Materials.

[9] Fair G.E., Kim H.J., Lee H., Parthasarathy T.A., Keller K.A., Miller Z.D. (2011). Development of ceramic fibers for high-energy laser applications. Laser Technol. Déf. Secur. VII.

[10] Gao P., Zhang L., Yao Q., Shao C., Wei S., Zhou T., Chen H., Yang H. (2020). Fabrication, mechanical and optical performance of AM-gel casted YAG transparent ceramics. Ceram. Int..

[11] Tian F., Chen C., Liu Y., Liu Q., Ivanov M., Wang Q., Jiang N., Chen H., Yang Z., Xie T. (2020). Fabrication of Nd:YAG transparent ceramics from co-precipitated powders by vacuum pre-sintering and HIP post-treatment. Opt. Mater..

[12] Parthasarathy T.A., Hay R.S., Fair G., Hopkins F.K. (2010). Predicted performance limits of yttrium aluminum garnet fiber lasers. Opt. Eng..

[13] Ikesue A., Aung Y.L., Taira T., Kamimura T., Yoshida K., Messing G.L. (2006). Progress in ceramic lasers. Annu. Rev. Mater. Res..

[14] Ikesue A., Aung Y.L., Okamoto T., Yamada K., Kamimura T., Yoshida K. Development of Free Designable Ceramic Fiber Lasers. Proceedings of the Conference on Lasers and Electro-Optics/Quantum Electronics and Laser Science Conference and Photonic Applications Systems Technologies.

[15] Kim H.J., Fair G.E., Hart A.M., Potticary S.A., Usechak N.G., Corns R.G., Hay R.S. (2015). Development of polycrystalline yttrium aluminum garnet (YAG) fibers. J. Eur. Ceram. Soc..

[16] Kim H.J., Fair G., Lee H., Keller K., Parthasarathy T.A., Hay R. (2011). Processing and transparency of polycrystalline yttrium aluminum garnet (YAG) fibers for optical applications. Solid State Lasers XX Technol. Dev..

[17] Kim H., Hay R.S., McDaniel S.A., Cook G., Usechak N.G., Urbas A.M., Shugart K.N., Lee H., Kadhim A.H., Brown D.P. (2017). Lasing of surface-polished polycrystalline Ho: YAG (yttrium aluminum garnet) fiber. Opt. Express.

[18] Gao P., Zhang L., Yao Q., Ma Y., Shao C., Zhou T., Liu M., Zhu L., Chen H., Cheng X. (2021). A novel route to fabricate Yb:YAG ceramic fiber and its optical performance. J. Eur. Ceram. Soc..

[19] Polisadova E., Valiev D., Vaganov V., Oleshko V., Han T., Zhang C., Burachenko A., Popov A. (2019). Time-resolved cathodoluminescence spectroscopy of YAG and YAG:Ce3+ phosphors. Opt. Mater..

[20] Dai Y., Zhang Z., Wang X., Lu Z., Kou H., Su L., Wu A. (2021). Growth and Characterization of Ce-Doped Luag Single Crystal Fibers from Transparent Ceramics by Laser-Heated Pedestal Method. Crystals.

[21] Amekura H., Li R., Okubo N., Ishikawa N., Chen F. (2020). Swift heavy ion irradiation to non-amorphizable CaF2 and amorphizable Y3Al5O12 (YAG) crystals. Nucl. Instrum. Meth. B.

[22] van Vuuren A.J., Saifulin M., Skuratov V., O’Connell J., Aralbayeva G., Dauletbekova A., Zdorovets M. (2018). The influence of stopping power and temperature on latent track formation in YAP and YAG. Nucl. Instrum. Meth. B.

[23] Kim H.J., Fair G.E., Hart A.M., Potticary S.A., Usechak N.G. (2012). Influence of processing variables on the properties of polycrystalline YAG fibers. Laser Technol. Déf. Secur. VIII.

[24] Lee H., Keller K., Sirn B., Parthasarathy T., Cheng M., Hopkins F.K. (2011). Recent developments in polycrystalline oxide fiber laser materials: Production of Yb-doped polycrystalline YAG fiber. Nanophotonics Macrophotonics Space Environ. V.

[25] Wang J., Yang B., Lin X., Gao L., Liu T., Lu Y., Wang R. (2020). Research of TPU Materials for 3D Printing Aiming at Non-Pneumatic Tires by FDM Method. Polymers.

[26] Gómez J., Recio I., Navas A., Villaro E., Galindo B., Ortega-Murguialday A. (2019). Processing influence on dielectric, mechanical, and electrical properties of reduced graphene oxide–TPU nanocomposites. J. Appl. Polym. Sci..

[27] Jia Z., Guo Z., Yuan C. (2021). Effect of Material Hardness on Water Lubrication Performance of Thermoplastic Polyurethane under Sediment Environment. J. Mater. Eng. Perform..

[28] Banoriya D., Purohit R., Dwivedi R.K. (2020). Wear performance of titanium reinforced biocompatible TPU. Adv. Mater. Process. Technol..

[29] Jiang Q., Liao X., Yang J., Wang G., Chen J., Tian C., Li G. (2020). A two-step process for the preparation of thermoplastic polyurethane/graphene aerogel composite foams with multi-stage networks for electromagnetic shielding. Compos. Commun..

[30] Lu Q.-W., Hernandez-Hernandez M.E., Macosko C.W. (2003). Explaining the abnormally high flow activation energy of thermoplastic polyurethanes. Polymer.

[31] Ke K., Bonab V.S., Yuan D., Manas-Zloczower I. (2018). Piezoresistive thermoplastic polyurethane nanocomposites with carbon nanostructures. Carbon.

[32] Chen J., Zhang Z.-X., Huang W.-B., Li J.-L., Yang J.-H., Wang Y., Zhou Z.-W., Zhang J.-H. (2015). Carbon nanotube network structure induced strain sensitivity and shape memory behavior changes of thermoplastic polyurethane. Mater. Des..

[33] Hausberger A., Major Z., Theiler G., Gradt T. (2018). Observation of the adhesive- and deformation- contribution to the friction and wear behaviour of thermoplastic polyurethanes. Wear.

[34] Sato S., Yamaguchi T., Shibata K., Nishi T., Moriyasu K., Harano K., Hokkirigawa K. (2020). Dry sliding friction and Wear behavior of thermoplastic polyurethane against abrasive paper. Biotribology.

[35] Mailhot B., Komvopoulos K., Ward B., Tian Y., Somorjai G.A. (2001). Mechanical and friction properties of thermoplastic polyurethanes determined by scanning force microscopy. J. Appl. Phys..

[36] Lu X., Qu J., Huang J. (2015). Mechanical, thermal and rheological properties of hollow glass microsphere filled thermoplastic polyurethane composites blended by normal vane extruder. Plast. Rubber Compos..

[37] Jaúregui-Beloqui B., Fernández-García J.C., Orgilés-Barceló A.C., Mahiques-Bujanda M.M., Martín-Martínez J.M. (1999). Rheological properties of thermoplastic polyurethane adhesive solutions containing fumed silicas of different surface areas. Int. J. Adhes. Adhes..

